# Identification of Novel Betaherpesviruses in Iberian Bats Reveals Parallel Evolution

**DOI:** 10.1371/journal.pone.0169153

**Published:** 2016-12-30

**Authors:** Francisco Pozo, Javier Juste, Sonia Vázquez-Morón, Carolina Aznar-López, Carlos Ibáñez, Inazio Garin, Joxerra Aihartza, Inmaculada Casas, Antonio Tenorio, Juan Emilio Echevarría

**Affiliations:** 1 Virology Section, Centro Nacional de Microbiología, Instituto de Salud Carlos III, Majadahonda, Madrid, Spain; 2 Estación Biológica de Doñana, CSIC, Seville, Spain; 3 Centro de Investigación Biológica en Red de Epidemiología y Salud Pública (CIBERESP), Madrid, Spain; 4 Department of Zoology and Animal Cell Biology, University of the Basque Country (UPV/EHU), Leioa, The Basque Country, Spain; University of Texas Medical Branch at Galveston, UNITED STATES

## Abstract

A thorough search for bat herpesviruses was carried out in oropharyngeal samples taken from most of the bat species present in the Iberian Peninsula from the *Vespertilionidae*, *Miniopteridae*, *Molossidae* and *Rhinolophidae* families, in addition to a colony of captive fruit bats from the *Pteropodidae* family. By using two degenerate consensus PCR methods targeting two conserved genes, distinct and previously unrecognized bat-hosted herpesviruses were identified for the most of the tested species. All together a total of 42 potentially novel bat herpesviruses were partially characterized. Thirty-two of them were tentatively assigned to the *Betaherpesvirinae* subfamily while the remaining 10 were allocated into the *Gammaherpesvirinae* subfamily. Significant diversity was observed among the novel sequences when compared with type herpesvirus species of the ICTV-approved genera. The inferred phylogenetic relationships showed that most of the betaherpesviruses sequences fell into a well-supported unique monophyletic clade and support the recognition of a new betaherpesvirus genus. This clade is subdivided into three major clades, corresponding to the families of bats studied. This supports the hypothesis of a species-specific parallel evolution process between the potentially new betaherpesviruses and their bat hosts. Interestingly, two of the betaherpesviruses’ sequences detected in rhinolophid bats clustered together apart from the rest, closely related to viruses that belong to the *Roseolovirus* genus. This suggests a putative third roseolo lineage. On the contrary, no phylogenetic structure was detected among several potentially novel bat-hosted gammaherpesviruses found in the study. Remarkably, all of the possible novel bat herpesviruses described in this study are linked to a unique bat species.

## Introduction

Herpesviruses make up a wide group of DNA viruses extensively disseminated in nature that is present in most vertebrates and, recently discovered, can even infect some marine bivalves. Due to the steady increase in the number of members recognized within this large group of viruses and their significant heterogeneity, the classification of herpesviruses has been recently updated by the International Committee on Taxonomy of Viruses (ICTV). The family *Herpesviridae* contains mammal, bird and reptile viruses. In addition, two other families have been created, one to harbor the fish and frog viruses (Family *Alloherpesviridae*), and another to include the herpesviruses found in invertebrate animals (Family *Malacoherpesviridae*), all three families grouped in the Order *Herpesvirales* [1]. Mammalian herpesviruses account for most of the diversity within *Herpesviridae* and represent ten out of the thirteen genera recognized to date. Within the mammalian group, primates have the highest number of described herpesviruses, grouped in six different genera. Ungulates and rodents also show a high number of diverse herpesviruses. They are distributed through several genera, three of which are exclusive. However, despite being increasingly recognized as common reservoir hosts for many viruses [2], bats have been relatively poorly studied in this aspect. In fact, and despite the remarkable accumulation of novel viruses associated with the improvement of new generation sequencing techniques, herpesviruses of bats are definitely underrepresented. Considering that the order *Chiroptera* contains 1,116 recognized species, represents around twenty per cent of the mammalian diversity world-wide and is the second most speciose mammal group after rodents [3], this is quite surprising.

The first reference to any herpesvirus detected in bats dates back to 1996 when cytomegalovirus-like particles were identified in the principal submandibular gland of the little brown bat (*Myotis lucifugus*) by electron microscopy [4]. Since then, herpesviruses, or herpesviral sequences, have been progressively documented in bat species. Evidence of alphaherpesviruses has recently been found in pteropid bats such as *Eidolon helvum*, *E*. *dupreanum*, *Pteropus lylei* (a nectarivorous bat), *Lonchophylla thomasi*, and an unidentified bat [5, 6]. Several betaherpesviruses and gammaherpesviruses have also been detected in bats from the families *Vespertilionidae* [7,8], *Miniopteridae* [9, 10], *Pteropodidae* [11], *Hipposideridae* [12] and *Rhinolophidae*[8]. Nevertheless, with the exception of a few vespertilionid gammaherpesviruses described by Wibbelt et al. [7], relatively little is known about the phylogenetic relationships among the different bat herpesvirus species.

Two consensus PCR methods targeting two well-conserved genes were designed in order to thoroughly study the viruses affecting bats in Iberia. The methods were especially designed with the aim of identifying and characterizing some, as of yet, unknown bat-associated herpesviruses and the relationships between them. Both methods were applied to oropharyngeal samples taken from most of the bat species known in the Iberian Peninsula. Samples from the *Vespertilionidae*, *Miniopteridae*, *Molossidae* and *Rhinolophidae* familes were taken. As a result, forty-two potentially novel bat herpesviruses are described in this study as well as their evolutionary relationships in relation to the available reference material.

## Materials and Methods

### Samples collection and handling

**Fig 1:**
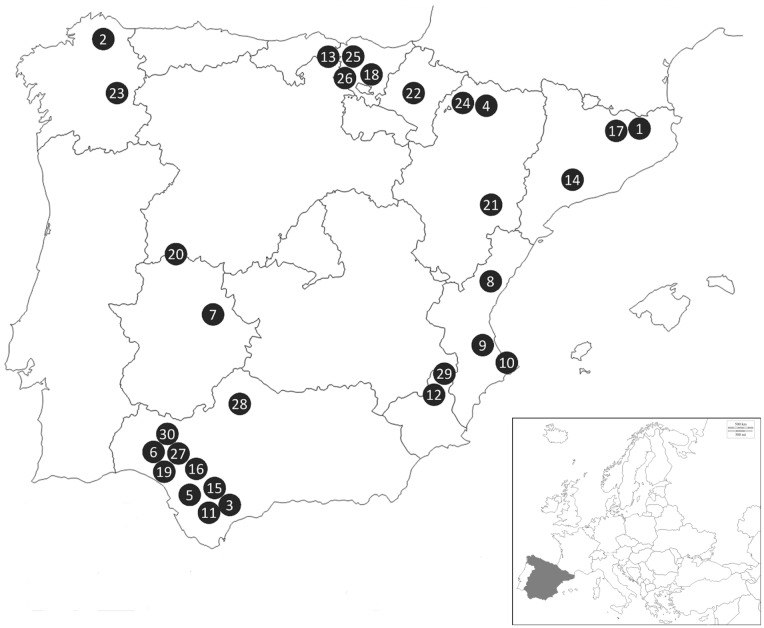
Bat capture sites in the Iberian Peninsula. 1 Albanyà, Girona. 2 As Pontes, A Coruña. 3 Benaoján, Málaga. 4 Boltaña, Huesca. 5 Bornos, Cádiz. 6 Calañas, Huelva. 7 Cañamero, Cáceres. 8 Castelló de la Plana, Castellón. 9 Cotes, Valencia. 10 Dénia, Alacant. 11 Gaucín, Málaga. 12 Jumilla, Murcia. 13 Karrantza, Bizkaia. 14 La Morera de Montsant, Tarragona. 15 Cortes de la Frontera, Málaga. 16 Las Cabezas de San Juan, Sevilla. 17 Montagut, Girona. 18 Mutiloa, Gipuzkoa. 19 Niebla, Huelva. 20 Nuñomoral, Cáceres. 21 Oliete, Teruel. 22 Pamplona, Navarra. 23 Samos, Lugo. 24 Santa Cruz de la Serós, Huesca. 25 Ugao, Bizkaia. 26 Valdegovía, Arava. 27 Villarrasa, Huelva. 28 Villaviciosa de Córdoba, Córdoba. 29 Yecla, Murcia. 30 Zalamea la Real, Huelva.

A total of 368 bats belonging to 26 species (Table 1) were captured and sampled through several campaigns in 30 different sites across the Iberian Peninsula from 2002 to 2008 (Fig 1). Sample collecting was completed as part of a bat rhabdoviruses and lyssaviruses surveillance program and was conducted according to the approved protocol by the General Research Program of the Spanish Government under the specific projects SAF2006-12784-C02-02 and SAF2009-09172. Collection permits were obtained from the respective Autonomous Comunities’ authorities. Collection methods followed the regulations and ethical procedures according to the Spanish Bat Society (SECEMU). Bats were mainly captured with mist-nets and hand-nets as they left diurnal roosts or along their nocturnal commuting flights. Bats were released at the same collecting point after being identified, measured, sexed and sampled. For cryptic species, taxonomic identification was confirmed genetically by amplification and sequencing of a diagnostic fragment of the mtDNA cytochrome B gene following Ibáñez et al. [13]. Additionally, 31 exotic African fruit bats (*Rousettus aegyptiacus*), from the *Pteropodidae* family, kept in captivity at the zoobotanical park of Jerez de la Frontera (Cádiz), were also sampled in 2010.

**Table 1:** Iberian bat species tested for herpesviruses.

Bat species			Oropharyngeal samples positive/total	Capture Location^a^	Capture Year
Family	Scientific name	Common name			
*Vespertilionidae*	*Barbastella barbastellus*	Barbastelle bat	0/4	1, 14	2007, 2008
	*Eptesicus isabellinus*	Meridional serotine bat	16/33	5, 12, 16, 19, 20, 27, 29, 30	2004, 2007
	*Eptesicus serotinus*	Serotine bat	11/15	2, 18, 25	2003, 2007
	*Hypsugo savii*	Savi’s pipistrelle	2/10	1, 17	2007
	*Myotis alcathoe*	Alcathoe’s bat	1/1	23	2007
	*Myotis bechsteinii*	Bechstein’s bat	2/3	1, 15	2007
	*Myotis blythii*	Lesser mouse-eared bat	6/6	10	2004
	*Myotis capaccinii*	Long-fingered bat	3/15	8, 9	2004
	*Myotis daubentonii*	Daubenton’s bat	10/26	3, 6, 7	2004, 2007
	*Myotis emarginatus*	Geoffroy’s bat	7/31	3, 28	2004, 2007, 2008
	*Myotis escalerai*	Iberian Natterer’s bat	9/17	1, 10, 17, 30	2004, 2007
	*Myotis myotis*	Greater mouse-eared bat	9/18	7, 9, 10	2004, 2007
	*Myotis mystacinus*	Whiskered bat	1/2	23	2007
	*Nyctalus lasiopterus*	Greater noctule bat	3/3	15	2007
	*Nyctalus leisleri*	Lesser noctule bat	6/8	1, 15, 17	2007
	*Nyctalus noctula*	Common noctule bat	13/18	22	2007
	*Pipistrellus kuhlii*	Kuhl’s pipistrelle	5/6	7, 15	2007
	*Pipistrellus pipistrellus*	Common pipistrelle	3/6	1	2007
	*Pipistrellus pygmaeus*	Soprano pipistrelle	1/1	7	2007
	*Plecotus austriacus*	Grey long-eared bat	9/11	1, 17, 12, 30	2004, 2007
*Miniopteridae*	*Miniopterus schreibersii*	Schreiber’s bat	29/40	7, 9, 10, 11, 15	2002, 2004, 2007
*Rinolophidae*	*Rhinolophus euryale*	Mediterranean horseshoe bat	0/52	4, 9, 28	2004, 2007, 2008
	*Rhinolophus ferrumequinum*	Greater horseshoe bat	15/24	3, 7, 12, 17, 28	2004, 2007, 2008
	*Rhinolophus hipposideros*	Lesser horseshoe bat	1/10	13, 20, 24, 26	2007, 2008
	*Rhinolophus mehelyi*	Mehely's horseshoe bat	0/1	7	2007
*Molossidae*	*Tadarida teniotis*	European free-tailed bat	6/7	21, 27	2008
*Pteropodidae*^b^	*Rousettus aegyptiacus*	Egyptian fruit bat	5/31		2010

^a^ See Fig 1 for details.

^b^ Exotic bat species in the Iberian fauna, kept captive in a zoo.

Oropharyngeal swabs were collected and preserved at room temperature in 1 mL of lysis buffer containing 4 M GuSCN (Sigma-Aldrich), 0.5% N-lauroylsarcosine (Sigma-Aldrich), 1 mM dithiothreitol (Sigma-Aldrich), 25 mM sodium citrate (Sigma-Aldrich) and 0.1 mg/mL glycogen (Boehringer Mannheim). Total nucleic acids were extracted from 200 μl of the buffered suspension of each swab, resuspended in 50 μL of high pure molecular biology grade water and then stored at -80°C until polymerase chain reaction (PCR) was performed.

### Panherpesvirus PCR methods

Two consensus PCR methods to detect all members of the *Herpesviridae* family targeting conserved regions of the exon 2 of the ATPase subunit of the terminase gene (panCSG) and the catalytic subunit of the DNA polymerase (panDPOL) were designed in a conventional nested format. Both methods were optimized with controlled DNA copies of human herpesviruses and two animal herpesviruses harboring a high guanine-cytosine content, the bovine herpesvirus 1 (BoHV1) and the suid herpesvirus 1 (SuHV1). The evaluation of both methods was done using clinical specimens containing human herpesviruses. The primers sets used are listed in Table 2.

**Table 2:** Degenerate primers sets used for amplification of novel bat herpesviruses.

PCR method	Primer^a^	Primer sequence (5’-3’)^b^	Genome position^c^	Amplicon size (bp)^d^
panCSG	CSGdeg1F	GTIGAYGARRSIMAYTTYAT	134873–134854	
	CSGdeg1R	TTKIIIGTRWAIGCIGGRTC	134389–134408	470–494
	CSGdeg2F	MYISYAARMTIATITTYRTITCITC	134782–134806	
	CSGdeg2R	GTRWAIGCIGGRTCIAIRTA	134395–134414	397–421
panDPOL	POLdeg1F	GAYTTYSMIAGYYTITAYCC	79774–79755	
	POLdeg1R	TTICKIACSARITCIACICCYTT	78969–78991	713–992
	POLdeg2F	ATIATIMWRGCICAYAAYYTITG	79750–79728	
	POLdeg2R	AAIAIISWRTCIGTRTCICCRTA	79179–79201	482–758

^a^ Forward (F) and reverse (R) strand for primer sequences.

^b^ Inosine (I) in the three- and four-fold degenerate positions.

^c^ Positions of primers in HHV5 reference genome (GenBank accession number NC_006273).

^d^ Amplicon size is variable depending on the virus detected.

All nucleic acids extracted from bat oropharyngeal samples (5 μL) were initially tested using a first round panCSG mixture containing 1 μM each of degenerate primers CSGdeg1F and CSGdeg1R, 0.4 mM each dNTP, 10% DMSO, 5% glycerol and 2.5 U of AmpliTaq DNA Polymerase (Applied Biosystems) in a buffer with a final concentration of 60 mM Tris–HCl (pH 8.5), 15 mM (NH_4_)_2_SO_4_ and 2 mM MgCl_2_ and adjusted for a total volume of 50 μL. The amplification conditions consisted of 2 min at 94°C, followed by 40 cycles for 30 sec at 94°C, 3 min at 40°C and 30 sec at 72°C, and a final extension at 72°C for 5 min in an automated PTC-200 Peltier Thermal Cycler (MJ Research). The second-round amplification was carried out adding 2 μL of primary amplification product to 48 μL of a secondary amplification mixture as above except for 0.2 mM each dNTP and 1 μM each of degenerate primers CSGdeg2F and CSGdeg2R under the same conditions used for the first round, except that the annealing temperature was 35°C. Amplification products were visualized by ethidium bromide staining following electrophoresis on 2% NuSieve (FMC BioProducts) agarose gels. Positive and negative PCR controls containing a fixed quantity of HHV5 genome and nuclease-free water were included in each run. Usual precautions were taken to avoid cross-contamination of samples before and after nucleic acids extraction and amplification. In order to increase phylogenetic accuracy, specimens with a positive result were subsequently subjected to a second method of nested PCR targeting the catalytic subunit of the DNA polymerase (panDPOL), using degenerate primers POLdeg1F and POLdeg1R for the first-round amplification and POLdeg2F and POLdeg2R for the second-round. The usual prevention measures to avoid false positive results due to cross-contamination were adopted. Briefly, a total of 5 μL was added to a reaction mixture containing 60 mM Tris–HCl (pH 8.5), 15 mM (NH_4_)_2_SO_4_, 2 mM MgCl_2_, 1 μM each of forward and reverse primers, 0.4 mM each dNTP, 5% DMSO and 2.5 U of AmpliTaq DNA Polymerase (Applied Biosystems) in a total volume of 50 μL. Amplification consisted of 1 cycle at 94°C for 2 min, followed by 40 cycles of 94°C for 30 sec, 45°C for 3 min, and 72°C for 30 sec, and a final extension at 72°C for 5 min. Two microliters of primary product were then transferred to 48 mL of the secondary amplification mixture as above except for 0.2 mM each dNTP. The samples were then incubated for 1 cycle at 94°C for 2 min and then 40 cycles of 94°C for 30 sec, 40°C for 3 min, and 72°C for 30 sec. Amplification products were visualized on a 2% agarose gel.

### Sequence analyses and data set alignments

Amplification products of the expected size were purified using QIAquick PCR Purification kit or QIAquick Gel Extraction kit (Qiagen) and directly double-strand sequenced by the Sanger chain-termination method using the BigDye Terminator v3.1 Cycle Sequencing Kit protocol and the ABI PRISM 3700 DNA Analyzer (Applied Biosystems). The nucleotide sequences of the amplification products excluding primer binding sites and the corresponding amino acid sequences were compared with those published in GenBank database using the BLASTn and BLASTp algorithms available on the National Center for Biotechnology Information webpage (http://blast.ncbi.nlm.nih.gov/). This procedure is sufficient to assess whether a virus is already known or novel and allows for a preliminary assignation of novel viruses to any of the herpesvirus subfamilies. As a first attempt to check for homology, amino acid sequence identity and similarity were calculated by global pairwise alignments with the homologous sequences of type herpesvirus species of the ICTV-approved genera using the EMBOSS Needle program (version 6.6.0), based on the Needleman-Wunsch algorithm and available at the European Bioinformatics Institute webpage (http://www.ebi.ac.uk/Tools/psa/emboss_needle/). This global alignment tool generates the best alignment over the entire length of each pair of sequences. The scoring parameters were fixed by using the amino acid substitution matrix BLOSUM62, and penalties for gaps: GOP = 15 and GEP = 0.5.

Multiple-sequence alignments of the nucleotide sequences obtained in this study and those retrieved from the GenBank database were performed using the online MAFFT software version 7.220 [14] and applying the E-INS-i algorithm, the 200PAM/k = 2 scoring matrix, a gap opening penalty of 1.53, and an offset value of zero (http://mafft.cbrc.jp/alignment/server/). Poorly aligned positions and highly divergent regions were removed using the online version of the GBlocks program [15] (http://molevol.cmima.csic.es/castresana/Gblocks_server.html), ensuring the selected blocks contained only complete codons and allowing smaller final blocks, some gap positions within the blocks and less strict flanking positions in order to get a less stringent selection.

Four different data sets (DS) were defined in the study, three of them containing aligned DNA sequences of viruses that belonged (or were assigned) to the *Betaherpesvirinae* subfamily. Data set 1 (DS1) contained sequences coming from the panCSG PCR method along with retrieved sequences from GenBank for this particular fragment. Alongside, data set 2 (DS2) comprised the panDPOL amplified fragment from our samples and retrieved sequences from GenBank. Data set 3 (DS3) was then constructed by concatenating DS1 and DS2, to yield more accurate reconstructions of the phylogenetic relationships. The gammaherpesvirus HHV4 was included as an outgroup in DS1-DS3 data sets. Another data set (DS4), was set up with the sequences of the panCSG PCR that belonged (or were assigned) to the *Gammaherpesvirinae* subfamily, again aligned with sequences retrieved from GenBank and including the human cytomegalovirus HHV5 as an outgroup.

### Phylogenetic inference

Phylogenetic reconstructions for all 4 data sets were obtained under Bayesian inference and using posterior probability criterion (BPP) implemented with the software MrBayes v3.2.1 [16]. Two simultaneous runs of 10^7^ generations were conducted for each data set respectively with 4 Markov chains, with a sampling frequency every 500 trees and assuming an initial burn-in of 25%. The best performing nucleotide substitution models for each data set (TrN+I+G for the terminase and GTR + I + G for the DNA polymerase fragments) were selected using the Bayesian information criteria (BIC) implemented in the program jModelTest [17]. Markov chain Monte Carlo (MCMC) sampling convergence was assessed by checking the average standard deviation of split frequencies dropped below 0.01. Convergence of chains was confirmed by the PSRF statistic implemented in MrBayes.

### Preliminary nomenclature, proposed abbreviations and GenBank accession numbers

The name of the viruses described in this study were assigned after their bat host species and by adding betaherpesvirus or gammaherpesvirus, depending on which subfamily the virus was tentatively assigned to and a number according to the sequential order of its finding. Abbreviations use the first letter of the generic host bat name and the first three letters of the specific host bat name, followed by suffixes BHV or GHV for betaherpesvirus or gammaherpesvirus (e.g., EisaBHV1 for *Eptesicus isabellinus* betaherpesvirus 1). The complete names, abbreviations and the corresponding GenBank accession numbers for the sequences of the potentially novel viruses described in this study are listed in Tables 3 and 4.

**Table 3:** Potentially novel bat betaherpesviruses.

Tentative virus name	Abbreviation	Positive^a^ / tested animals	GenBank Accession n°		% Amino acid sequence identity^b^			
			terminase	polymerase	HHV5	MuHV1	EEHV1	HHV6A
Eptesicus isabellinus betaherpesvirus 1	EisaBHV1	14/33	JX294544	KT886843	75.4	77.0	49.2	59.0
Eptesicus isabellinus betaherpesvirus 2	EisaBHV2	1/33	JX294545	KR608281	74.6	76.2	49.2	59.0
Eptesicus serotinus betaherpesvirus 1	EserBHV1	11/15	JX294546		75.4	77.0	49.2	59.0
Hypsugo savii betaherpesvirus 1	HsavBHV1	2/10	JX294547	KR608282	74.6	74.6	50.0	59.0
Miniopterus schreibersii betaherpesvirus 1	MschBHV1	1/40	EF151197		70.5	74.6	49.2	58.2
Miniopterus schreibersii betaherpesvirus 2	MschBHV2	26/40	EF151196	KR608283	69.7	74.6	49.2	59.0
Miniopterus schreibersii betaherpesvirus 3	MschBHV3	1/40	JX294548		62.0	66.7	45.4	51.9
Myotis alcathoe betaherpesvirus 1	MalcBHV1	1/1	JX294552	KR608287	73.0	75.4	49.2	57.4
Myotis bechsteinii betaherpesvirus 1	MbecBHV1	2/3	JX294549		73.1	76.5	48.7	56.3
Myotis blythii betaherpesvirus 1	MblyBHV1	6/6	EF151194		74.6	73.8	50.0	54.1
Myotis daubentonii betaherpesvirus 1	MdauBHV1	9/26	JX294550	KR608284	73.8	76.2	49.2	58.2
Myotis emarginatus betaherpesvirus 1	MemaBHV1	4/31	JX294551	KR608285	73.0	74.6	48.4	56.6
Myotis escalerai betaherpesvirus 1	MescBHV1	8/17	EF151193		74.6	74.6	50.8	55.7
Myotis escalerai betaherpesvirus 2	MescBHV2	1/17	KT886845	KT886844	73.0	75.4	49.2	57.4
Myotis myotis betaherpesvirus 1	MmyoBHV1	8/18	EF151195	KR608286	74.6	73.8	50.0	54.1
Myotis mystacinus betaherpesvirus 1	MmysBHV1	1/2	JX294553	KR608288	73.0	74.6	49.2	58.2
Nyctalus lasiopterus betaherpesvirus 1	NlasBHV1	2/3	JX294554	KR608289	76.2	77.9	50.0	60.7
Nyctalus leisleri betaherpesvirus 1	NleiBHV1	5/8	JX294555	KR608290	73.0	74.6	50.0	58.2
Nyctalus noctula betaherpesvirus 1	NnocBHV1	13/18	JX294556		75.4	77.0	48.4	59.8
Pipistrellus kuhlii betaherpesvirus 1	PkuhBHV1	5/6	JX294557	KR608291	75.4	77.0	49.2	60.7
Pipistrellus pipistrellus betaherpesvirus 1	PpipBHV1	2/6	JX294558		75.4	76.2	49.2	59.8
Pipistrellus pipistrellus betaherpesvirus 2	PpipBHV2	1/6	JX294559	KR608292	74.6	76.2	50.0	59.8
Pipistrellus pygmaeus betaherpesvirus 1	PpygBHV1	1/1	JX294560		74.6	75.4	49.2	59.0
Plecotus austriacus betaherpesvirus 1	PausBHV1	8/11	JX294561	KR608293	73.0	74.6	50.8	59.0
Plecotus austriacus betaherpesvirus 2	PausBHV2	1/11	JX294562		73.0	74.6	50.0	58.2
Rhinolophus ferrumequinum betaherpesvirus 2	RferBHV2	15/24	JX294567	KR608294	62.3	62.3	46.7	76.2
Rhinolophus hipposideros betaherpesvirus 1	RhipBHV1	1/10	JX294568		66.4	63.1	50.4	78.7
Rousettus aegyptiacus betaherpesvirus 1	RaegBHV1	1/31	JX294565		66.4	73.0	47.5	53.3
Rousettus aegyptiacus betaherpesvirus 2	RaegBHV2	1/31	JX294566		64.8	73.0	48.4	52.5
Tadarida teniotis betaherpesvirus 1	TtenBHV1	2/7	JX294563	KR608295	70.5	76.2	48.4	54.9
Tadarida teniotis betaherpesvirus 2	TtenBHV2	2/7	JX294564	KR608296	68.9	74.6	47.5	55.7
Tadarida teniotis betaherpesvirus 3	TtenBHV3	1/7		KR608297				

^a^ Number of positive animals in the PCR method targeting the terminase gene

^b^ Related to terminase. Viruses used for comparison were Human herpesvirus 5 (HHV5), Murid herpesvirus 1 (MuHV1), Elephant endotheliotropic herpesvirus 1 (EEHV1) and Human herpesvirus 6 strain U1102 (HHV6A).

**Table 4:** Potentially novel bat gammaherpesviruses.

Tentative virus name	Abbreviation	Positive^a^ / tested animals	GenBank Accession n°		% Amino acid sequence identity^b^			
			terminase	polymerase	HHV4	AlHV1	EHV2	SaHV2
Eptesicus isabellinus gammaherpesvirus 1	EisaGHV1	1/33	KR608273		63.7	52.4	55.2	53.2
Miniopterus schreibersii gammaherpesvirus 1	MschGHV1	1/40	KR608278	KR608298	59.7	66.1	67.2	72.6
Myotis capaccinii gammaherpesvirus 1	McapGHV1	3/15	KR608274		60.5	59.7	64.8	64.5
Myotis daubentonii gammaherpesvirus 1	MdauGHV1	1/26	KR608275		52.4	48.4	47.2	43.5
Myotis emarginatus gammaherpesvirus 1	MemaGHV1	3/31	KR608276		54.0	51.6	47.2	43.5
Myotis myotis gammaherpesvirus 1	MmyoGHV1	1/18	KR608277		59.7	59.7	64.0	63.7
Nyctalus lasiopterus gammaherpesvirus 1	NlasGHV1	1/3	KR608279		58.1	58.9	67.2	64.5
Nyctalus leisleri gammaherpesvirus 1	NleiGHV1	1/8		KR608299				
Rousettus aegyptiacus gammaherpesvirus 1	RaegGHV1	3/31	KR608280		54.0	55.6	68.8	67.7
Tadarida teniotis gammaherpesvirus 1	TtenGHV1	1/7		KR608300				

^a^ Number of positive animals in the PCR method targeting the terminase gene

^b^ Related to terminase. Viruses used for comparison were Human herpesvirus 4 (HHV4), Alcelaphine herpesvirus 1 (AlHV1), Equid herpesvirus 2 (EHV2) and Saimiriine herpesvirus 2 (SaHV2).

### Nomenclature, acronyms and GenBank accession numbers of published viruses

Updated formal taxonomic names and the corresponding acronyms used in this study follow the recommendations of the *Herpesviridae* Study Group of the International Committee on Taxonomy of Viruses (ICTV) [1] and are presented as supporting information”S1 File”.

## Results

Out of the total 368 Iberian bats studied, herpesviral genomic sequences were detected in 168 (45%) of the samples, which were all confirmed by sequencing. DNA of at least one herpesvirus was detected in 23 out of the 26 Iberian bat species analyzed, and also in the fruit bat *Rousettus aegyptiacus* (Table 1). Only the bats *Barbastella barbastellus* (0/4), *Rhinolophus mehelyi* (0/1) and *R*. *euryale* (0/52) failed to show evidence of herpesviruses in their oropharynx sampling. The presence of at least two different herpesviruses in the same specimen was detected in six oropharyngeal swabs which subsequently were not further analyzed. A total of 42 different herpesviruses were detected, most of them using the panCSG PCR (39/42), with 19 of them rendering a positive result also in a second PCR method targeting the DNA polymerase (panDPOL).Three herpesviruses were only identified by the panDPOL PCR method. Novel herpesvirus sequences were tentatively assigned to the *Betaherpesvirinae* (32/42) and *Gammaherpesvirinae* subfamilies (10/42). No evidence of alphaherpesviruses was found in the saliva of the bats sampled. Remarkably, every single herpesvirus species was linked to a specific bat species. However, detection of several herpesviruses per bat species was a common finding, with 12 species hosting two or more herpesviruses. The bats *Miniopterus schreibersii* and *Tadarida teniotis* contributed the most to the list of novel herpesvirus sequences detected. Each showed four different viruses, three betaherpesviruses and one gammaherpesvirus. For the purpose of this report all potentially novel herpesviruses were tentatively named, as described in Materials and Methods and listed with GenBank accession numbers in Tables 3 and 4.

Pairwise sequence comparisons with the type herpesvirus species of the ICTV-approved genera showed a range of distinct percent amino acids identity values in the target terminase. This depended on the genus considered. Bat herpesviruses assigned to the *Betaherpesvirinae* subfamily presented ranges between 62–76% identity when compared with HHV5, the type herpesvirus species of the *Cytomegalovirus* genus, 62–78% identity with MuHV1 (*Muromegalovirus*), 45–51% identity with EEHV1 (*Proboscivirus*), 52–61% identity with HHV6A (*Roseolovirus*). The last one, with the exception of the herpesviruses detected in the two rhinolophid bats that showed much higher identities with the *Roseolovirus* (76% and 79% respectively). Regarding the bat herpesviruses ascribed to the *Gammaherpesvirinae* subfamily, values ranged between 52–64% when compared with HHV4 (*Lymphocryptovirus*), 48–66% with AlHV1 (*Macavirus*), 47–69% with EHV2 (*Percavirus*), and 43–73% with SaHV2 (*Rhadinovirus*).

Data set DS1 consisted in an alignment of 52 homologous sequences of a 373 bp long fragment of the terminase gene including 31 novel bat herpesvirus sequences and 21 sequences retrieved from GenBank. Similarly, data set DS2 consisted in an alignment of 50 sequences of a 412 bp long fragment of the DNA polymerase gene and being 19 of them novel bat herpesvirus sequences. Data set DS3 was constituted by concatenating both the terminase and the DNA polymerase fragments. This came to a total of 785 aligned characters for 40 betaherpesviruses. Finally, data set DS4 consisted of an alignment of 43 sequences of a 376 bp long fragment of the terminase gene, including 9 novel bat herpesvirus sequences and 34 retrieved from GenBank.

**Fig 2:**
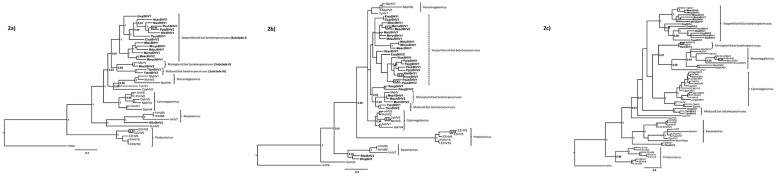
Phylogenetic analysis of potentially novel bat betaherpesviruses. Phylogenetic relationships of the novel bat-hosted betaherpesviruses (in bold) with their arrangement in relation to the main groups of betaherpesviruses available from GenBank. The reconstructions were built under the Bayesian criterion allowing specific model rates. The consensus topologies show Bayesian posterior probabilities (BPP) >0.7 after sampling 10^7^ generations. The phylogenetic analysis was based on the alignments of **2a)** the concatenated terminase (373bp) and polymerase (412 bp) fragments. **2b)** the fragment of the ATPase subunit of the terminase gene. **2c)** the fragment of the conserved region of the catalytic subunit of the DNA polymerase gene. Abbreviations of full virus names follow the designation explained in Materials and Methods and presented in the supporting information “S1 File”.

The phylogenetic analysis performed with the DS3 alignment (Fig 2a) showed that, with the exception of those detected in rhinolophid bats, all bat herpesvirus sequences tentatively assigned to the *Betaherpesvirinae* subfamily grouped in a well-supported monophyletic clade. This clade was subsequently subdivided into three well-supported subclades: Subclade I comprising of all novel viruses detected in vespertilionid bats (EisaBHV1, EisaBHV2, HsavBHV1, MalcBHV1, MdauBHV1, MemaBHV1, MescBHV2, MmyoBHV1, MmysBHV1, NlasBHV1, NleiBHV1, PausBHV1, PkuhBHV1 y PpipBHV2); subclade II comprising of viruses detected in miniopterid bats (novel virus MschBHV2 and the previously described virus MsHV [10], both found in *Miniopterus schreibersii*); and subclade III comprising of novel viruses detected in molossid bats (TtenBHV1 and TtenBHV2). Furthermore, subclade I visibly branched into two different groups, one containing viruses detected in bats belonging to the genus *Myotis*, and a second group containing viruses linked to the rest of the vespertilionid bats. Phylogenetic analysis performed using the individual gene fragments (DS1 and DS2 alignments) presented lower resolution than the combination of both (Fig 2b and 2c), showing DS2 in general in a better resolution than DS1. Nevertheless, these separated data sets allowed for the inclusion of more novel virus sequences as well as more additional reference material in the analyses that, in turn, have helped clarifying the hypothesized phylogenetic relationships. For instance, terminase-based phylogeny (DS1) has enabled the inclusion of the novel viruses RaegBHV1 and RaegBHV2 found in the fruit bat *Rousettus aegyptiacus* that grouped together in a separate clade but linked to other bat viruses (Fig 2b). Besides, the DS1-based phylogeny has allowed the addition of the novel viruses EserBHV1, MbecBHV1, MblyBHV1, MescBHV1, NnocBHV1, PausBHV2, PpipBHV1 and PpygBHV1, which are all found in vespertilionid bats. Additionally, the novel viruses MschBHV1 and MschBHV3 that are detected in *Miniopterus* schreibersii, have extended subclades I and II respectively. Remarkably, the newly added novel virus detected in *Rhinolophus hipposideros* (RhipBHV1) clustered together with the virus RferBHV2 from *R*. *ferrumequinum* in a unique group separated from all other bat herpesviruses related to the viruses that compose the *Roseolovirus* genus (Fig 2b). On the other hand, the polymerase-based phylogeny (DS2) has allowed the inclusion of the already known TrBHV1 virus detected in the vespertilionid bat *Tylonycteris robustula* in China [8] and that interestingly, appears closely related to viruses linked to the bat genus *Myotis*. Moreover, this phylogeny has allowed for the inclusion of the already described virus BatBHV2 detected in *Miniopterus fuliginosus* in Japan [9] and the novel virus TtenBHV3 found in *Tadarida teniotis* that have given support to the defined subclade II and subclade III respectively (Fig 2c). Finally, the inclusion of the known virus RfBHV1 from a *R*. *ferrumequinum* detected in China [8] has reinforced the remarkable association found between our novel viruses detected in *Rhinolophus* bats and the genus *Roseolovirus*.

Regarding to the bat herpesviruses tentatively placed in the *Gammaherpesvirinae* subfamily, only one (MschGHV1), out of the ten potentially novel gammaherpesviruses found was positive for both markers. Consequently, the phylogenetic analysis relied solely on the alignment of the 376 bp fragment of the exon 2 of the ATPase subunit of the terminase (DS4). This brought about quite low resolution and few nodes of the resulting topology were actually well-supported (See supporting information “S2 File”).

## Discussion

Within this study, bat herpesviruses have been largely examined across 26 out of the 31 bat species found so far in the Iberian Peninsula, covering the four families of bats recognized at present in Europe (*Vespertilionidae*, *Miniopteridae*, *Molossidae* and *Rhinolophidae*). Forty-two potentially novel bat herpesviruses were identified, most of them (32 out of 42) tentatively assigned to the *Betaherpesvirinae* subfamily and the remaining 10 allocated into the *Gammaherpesvirinae* subfamily. The high proportion of betaherpesviruses found can be explained by the fact that only oropharyngeal swabs were tested. It is well-known that betaherpesviruses can be maintained in latency in secretory glands representing a major site of betaherpesviruses replication and transmission [18]. In particular, salivary glands have been shown to represent a privileged site for cytomegalovirus immune evasion and persistence [19]. No alphaherpesviruses were detected in this study nor in the study by Wibbelt et al. [7] who analyzed bat lung tissues. However, evidence of alphaherpesviruses in other bats has recently been provided from spleen tissues of Indonesian fruitbats [5], and from throat swabs and salivary glands of other fruit bats from the genera *Eidolon* and *Pteropus* [6]. The absence of alphaherpesviruses in bats aside from fruit bats could indicate that bat alphaherpesviruses evolved only in the *Pteropodidae* family. Nevertheless, and according to the supra-family relationships described recently among bats [20], we would expect them to also appear in the other families, such as, *Rhinolophidae* that form, together with the pteropodids, the recently recognized *Yinpterochiroptera* suborder. Although we analyzed oropharyngeal specimens from 31 captive *Rousettus aegyptiacus*, this sampling could not have been enough to successfully detect the alphaherpesviruses. Another possible explanation is that the primers used for the universal detection of herpesviruses were less effective for detecting viruses belonging to the subfamily *Alphaherpesvirinae*, but this is not likely since the design and optimization processes of the panherpesvirus PCR were carried out with representative viruses of genera *Simplexvirus*, *Varicellovirus*, *Iltovirus* and *Mardivirus*, among others.

The detection of herpesviruses was carried out by amplifying two genomic regions included in the core genes of herpesviruses, being two out of the only three detectably conserved genes in the order *Herpesvirales* [21]. As a consequence, the terminase gene has previously been chosen to describe alpha- [22, 23], beta- [24] and gammaherpesviruses [25–28] in an assorted representation of animals. Likewise, the catalytic subunit of the DNA polymerase, first used by Van Devanter et al. [29] and then improved by Ehlers et al. [30], has been employed in a myriad of studies describing novel members of the three subfamilies of the family *Herpesviridae*, making this gene the most frequently sequenced of the herpesviral genome. Both PCR methods were subjected to a thorough optimization process, nevertheless the panDPOL method did not reach the sensitivity of that designed in the terminase gene, probably because the amplified fragment was much longer. Still, this marker was highly informative and contributed significantly to the high level of phylogenetic resolution obtained when concatenating the two fragments.

An important amount of bats out of the total analyzed (45%) harbored at least one herpesvirus at the time of sampling revealing, in general, a wide distribution for most of bat herpesviruses and a high frequency of reactivation. Because bats were captured along different years and at different locations, the possibility that viruses of the same bat species were originated from the same roost population was ruled out. Even so, the percentage of positives for this study is slightly lower than the 60% found in the only other comprehensive specific search for herpesviruses in bats published to date, that was carried out in 25 bats from eight vespertilionid species [7]. Nevertheless, some particular species, such as *Eptesicus serotinus* (11/15; 73%), *Miniopterus schreibersii* (29/40; 72%) and *Rhinolophus ferrumequinum* (15/24; 62%) showed higher percentages of positivity in our study. On the contrary, we did not detect any herpesvirus in the species *Rhinolophus euryale*, despite sampling a total of 52 individuals along three years and from different sites. This difference in prevalence for the two horse-shoe bats is quite surprising since both *Rhinolophus* are quite close both phylogenetically and ecologically, and in fact, is quite usual to find them sharing roosts.

Every possible novel bat herpesvirus described in this study was linked to a unique bat species. This species-specificity is a common feature for most of the viruses belonging to *Beta-* and *Gammaherpesvirinae* subfamilies [31]. In contrast to this, the study carried out by Wibbelt et al. showed infection of different hosts with the same herpesvirus [7]. The authors detected five out of the eight described viruses in more than one bat species, even belonging to different bat genera. The virus BatGHV-1 was the most remarkable case, being detected in four different bats (*Eptesicus serotinus*, *Myotis nattereri*, *Pipistrellus pipistrellus* and *P*. *nathusii*). Razafindratsimandresy et al. also reported the detection of the same herpesvirus in two bats of the same genus *Eidolon*, with distinct distribution areas [6]. On the other hand, our study shows that more than one herpesvirus could be hosted by same bat species. Nearly half of the bats tested (12/27) were associated with two or more herpesviruses, being the species *Miniopterus schreibersii* and *Tadarida teniotis* the most important contributors to the list of novel herpesviruses detected with four different viruses. This is not surprising, since it is well-known that vertebrate often host more than one herpesvirus. Although, some species are particularly noteworthy because of the number of viruses they can host. For example, humans, with eight different herpesviruses known to date, the rodent *Bandicota indica*, can also harbor eight viruses, four betaherpesviruses and four gammaherpesviruses [32], and the common chimpanzee *Pan troglodytes*, presenting five different cytomegaloviruses [33].

Phylogenetic relationships within the novel bat herpesvirus sequences detected in the Iberian bats tentatively assigned to the *Betaherpesvirinae* subfamily showed that most of them group in a monophyletic clade, further subdivided into three well-supported subclades matching three of the families of bats included in the study (*Vespertilionidae*, *Miniopteridae* and *Molossidae*). Our results are largely congruent and sustain the hypothesis of a parallel evolution of herpesviruses and their natural host species [34]. Besides the main subdivisions, our phylogenetic reconstructions show a deep split into a clade corresponding to the betaherpesviruses found in the bat genus *Myotis* and a clade containing the rest of the viruses found in the rest of the vespertilionid bats. This subdivision is again matching the phylogenetic reconstruction of the evolutionary relationships of bats. In fact, the genus *Myotis* shows both morphological and molecularly unique characteristics. Despite having been included traditionally in the subfamily *Vespertilioninae*, they are at present allocated their own subfamily *Myotinae* [35]. Furthermore, the supported internal grouping of the viruses found in the large *Myotis myotis* (MmyoBHV1) and in *M*. *escalerai* (MescBHV2) against the rest of the Eurasian *Myotis*, mirrors again the evolutionary reconstruction within the genus *Myotis* that separates this clade [36]. Likewise, the analysis clearly distinguishes the two viruses hosted by the two cryptic species *M*. *mystacinus* (MmysBHV1) and *M*. *alcathoe* (MalcBHV1) that were only recently separated with molecular techniques [37]. Interestingly, the already known virus TrBHV1 found in the Asian vespertilionid *Tylonycteris robustula* clusters into the *Myotis* group in the DS2-based phylogenetic analysis. This may represent a rare event of spill-over between bats, although a more comprehensive analysis, including other parts of the genome and taxa, are needed to have a more complete picture of this relationship. Another well-supported cluster includes the potentially novel viruses NlasBHV1, NleiBHV1, PpipBHV2 and PkuhBHV1, whose specific hosts are all bats belonging to the well-differentiated tribe *Pipistrellini* within the vespertilionid bats.

Remarkably, the new viruses RferBHV2 and RhipBHV1, detected in *Rhinolophus ferrumequinum* and *R*. *hipposideros* respectively, cluster together with the already known virus RfBHV1 detected in another *R*. *ferrumequinum* captured in China in a clade clearly apart from all the other bat betaherpesviruses, and closely related to viruses belonging to the genus *Roseolovirus*. The internal topology showed by our phylogenetic reconstruction within this clade is in accordance with the distinction of two *Roseolovirus* lineages already described by Staheli *et al*. [38]. One, containing the human viruses HHV6A and HHV6B, and the primate homologs PanHV6 and MneHV6 (provisionally termed roseolo1 lineage), and the second group, that includes the human virus HHV7 and their simian homologs MneHV7, PtroHV7, PpanHV7 and GgorHV7 (provisionally termed roseolo2 lineage). A putative third roseolo lineage would be constituted by the bat-hosted herpesviruses, linked to the base of the *Roseolovirus* clade within a long phylogenetic branch and with relatively low amino acid sequence identity when compared with the type virus HHV6A (Table 3). RferBHV2, RhipBHV1 and RfBHV1 would represent the only non-primate roseolo viruses reported to date, together with SuHV2. This virus is frequently known as porcine cytomegalovirus and shows in our reconstructions an uncertain position according to the individual markers but it has been recently claimed as a member of the genus *Roseolovirus* [39] as it appears in our combined phylogeny. Interestingly, rhinolophid bats are the only hosts for these viruses where no other virus belonging to the sister group of bat betaherpesviruses has been detected on them. Again, this finding is in total congruence with the evolutionary history of bats. In fact, rhinolophids show unique morphological and functional characteristics (eg. unique Doppler based echolocation system) and recent classifications [40] support the hypothesis that they branched off from the rest of *Chiroptera* at the origin of the group’s diversification, back in the Eocene about 50 million years ago [41]. Together with the fruit bats *Pteropodidae*, rhinolophids constitute along with their closest relatives families of echolocating bats (*Hipposideridae*, *Megadermatidae*, *Rhinopomatidae* and *Craseonycteridae*), the distinct suborder *Yinpterochiroptera*. Therefore, it would not be a surprise to find other roseolo-related viruses within this group of bat families too.

In relation to the *Pteropodidae* family, and despite the fact that we could only recover their partial sequences of the terminase gene, the possible novel betaherpesviruses RaegBHV1 and RaegBHV2 found in the fruit bat *Rousettus aegyptiacus* cluster apart from the other viruses in our phylogenetic reconstruction. This suggests a possible new group corresponding to the fruit bats family. Nevertheless, this point needs confirmation with extra sampling or additional markers. Interestingly, these fruit bats viruses fall within the rest of bat-hosted betaherpesviruses and do not group near the roseolo-related viruses as it would be expected according to the evolutionary relationships of bats. Our topologies also connect the miniopterid bat betaherpesviruses (subclade II) as a sister group of the molossid bat betaherpesviruses (subclade III) when it is known that *Miniopteridae* bats are more related to the *Vespertilionidae* [42]. This is probably just an apparent contradiction since all three bat families are actually closely related. The inclusion of viruses from a wider representation of the extant nineteen bat families will surely help clarify the evolutionary relationships between the groups of betaherpesviruses. In fact, this study has shown that the phylogenic reconstruction of the beherpesviruses retrieves families, many of the major groups within families, and even relationships at the intra-generic level known among bats. However, an issue that is still open is the question to what extent the relationships between bat-hosted betaherpesviruses will match the evolutionary relationships between the bats themselves. In any case, our phylogenies indicate that at least two different transfer events occurred in the evolutionary story of bat betaherpesviruses and at the origin of bats diversification. Since then, speciation events have followed those of their bat hosts with very few successful spill-overs between host species.

Unfortunately, the inference of phylogenetic relationships between the bat herpesviruses tentatively placed in the *Gammaherpesvirinae* subfamily was very limited because the sequences obtained of the fragment of the DNA polymerase -the most used gene in novel herpesviruses description- were too short for a reliable analysis. Still, several possible novel viruses were partially characterized, and the alignment of the terminase gene allowed some hypothesis about their phylogenetic relationships. The topology, nevertheless, was poorly solved and the bat-hosted novel viruses were scattered, without showing any monophyletic grouping along the shallow structure found in the tree, even at a family level. These results could be expected since it is well known that the *Gammaherpesvirinae* is the most complex of the three subfamilies of mammalian herpesviruses. In fact, *Gammaherpesvirinae* presents a high number of distinct deep lineages, particularly among the viruses belonging to the genus *Rhadinovirus* which apparently do not belong to any of the currently defined genera [43]. To further illustrate the complexity of the gammaherpesviruses phylogenetic relationships, different divergent lineages of viruses have also been found in other mammalians orders, such as *Primates* [44], or *Artiodactyla* [45, 46], indicating that gammaherpesviruses and their hosts evolved in a much more loose and complex way than other herpesviruses.

All in all, the significant number of possible novel bat herpesviruses described in the present study adds important information to our understanding of the evolution of the herpesviruses, particularly, to the *Betaherpesvirinae* subfamily. Specifically, our findings support the recognition of a new genus comprising all betaherpesviruses found in bats, assuming that new characterization data will be further available in the future to come. Additionally, and based on the topologies provided, bat betaherpesviruses seem to have evolved in a species-specific parallel evolution mode together with their hosts.

## Supporting Information

S1 File Nomenclature, acronyms and GenBank accession numbers of published viruses. (DOCX)

S2 File Phylogenetic relationships of the potentially novel bat-hosted gammaherpesviruses. (DOCX)
