# Supplementary material for: Identification of Novel Betaherpesviruses in Iberian Bats Reveals Parallel Evolution
Source: PLoS One. 2016 Dec 30;11(12):e0169153. doi: 10.1371/journal.pone.0169153 (PMC5201282; doi:10.1371/journal.pone.0169153)
Supplement: S1 File — (DOCX) [file pone.0169153.s001.docx]

**Supporting information (S1)**

**Nomenclature, acronyms and GenBank accession numbers of published viruses**

For sequence alignment and phylogenetic analyses all the subsequent herpesvirus sequences of required size available in the GenBank database were used, namely: **(i) Genus *Cytomegalovirus*:** Aotine herpesvirus 1, AoHV1 (complete genome [cg], accession number NC_016447); Cebine herpesvirus 1, CbHV1 (DNA polymerase [pol], JQ264772); *Cercocebus agilis* cytomegalovirus 1, CagiCMV1 (pol, AY608713); Cercopithecine herpesvirus 5, CeHV5 (cg, NC_012783); *Cercopithecus cephus* cytomegalovirus 1, CcepCMV1 (pol, AY728178); *Colobus guereza* cytomegalovirus 1, CgueCMV1 (pol, AY129397); *Gorilla gorilla* cytomegalovirus 2, GgorCMV2 (pol, FJ538490); Human herpesvirus 5, HHV5 (cg, NC_006273); Macacine herpesvirus 3, McHV3 (cg, NC_006150); *Mandrillus leucophaues* cytomegalovirus, MndCMV (pol, AF282941); *Mandrillus leucophaues* cytomegalovirus strain OCOM6-2, DrCMV (pol, AF387665); *Mandrillus sphinx* cytomegalovirus, MsphCMV1 (pol, AY129399); Panine herpesvirus 2, PnHV2 (cg, NC_003521); Papiine herpesvirus 3, PaHV3 (pol, AF387664); *Pongo pygmaeus* cytomegalovirus 1, pongoCMV1 (pol, AY129396); Saimiriine herpesvirus 4, SaHV4 (cg, NC_016448). **(ii) Genus *Muromegalovirus*:** *Apodemus flavicollis* cytomegalovirus 2, AflaCMV2 (pol, EF125063); *Bandicota indica* cytomegalovirus 3, BindCMV3 (pol, EF125067); *Microtus arvalis* cytomegalovirus 1, MarvCMV1 (pol, EF125059); Murid herpesvirus 1, MuHV1 (cg, NC_004065); Murid herpesvirus 2, MuHV2 (cg, NC_002512); Murid herpesvirus 8, MuHV8 (cg, NC_019559); *Mus musculus* cytomegalovirus 2, MmusCMV2 (pol, GU017485); *Myodes glareolus* cytomegalovirus 1, MglaCMV1 (pol, EF125061); *Rattus exulans* cytomegalovirus 1, RexuCMV1 (pol, EF125071). **(iii) Genus *Roseolovirus*:** *Gorilla gorilla* herpesvirus 7, GgorHV7 (pol, KJ843243); Human herpesvirus 6 strain U1102, HHV6A (cg, NC_001664); Human herpesvirus 6 strain Z29, HHV6B (cg, NC_000898); Human herpesvirus 7, HHV7 (cg, NC_001716); *Macaca nemestrina* herpesvirus 6 and 7, MneHV6 and MneHV7 (partial polymerase nucleotide sequences kindly provided by Jeannette Staheli, Center for Global Infectious Disease Research at Seattle Children's Research Institute); Mandrillus herpesvirus beta, MndHVβ (pol, AF282942); *Pan paniscus* herpesvirus 7, PpanHV7 (pol, KJ843241); *Pan troglodytes* herpesvirus 6, panHV6 (pol, AY359407); *Pan troglodytes* herpesvirus 7, panHV7 (pol, KJ843244). **(iv) Genus *Proboscivirus*:** Elephant endotheliotropic herpesvirus 2, EEHV2 (pol, HM568558; ATPase subunit of terminase [ter], JQ300040); Elephant endotheliotropic herpesvirus 3, EEHV3 (pol, JQ300065); Elephant endotheliotropic herpesvirus 4, EEHV4 (pol, EU658934); Elephant endotheliotropic herpesvirus 5, EEHV5 (cg, NC_024696); Elephant endotheliotropic herpesvirus 6, EEHV6 (pol, HM060765; ter, JQ300061); Elephant endotheliotropic herpesvirus 7, EEHV7 (pol, JQ300083); Elephantid herpesvirus 1 strain Raman, ElHV1A (cg, NC_020474); Elephantid herpesvirus 1 strain Emelia, ElHV1B (cg, KC462164). **(v) Unassigned species in the *Betaherpesvirinae* subfamily:** Bat betaherpesvirus 2, BatBHV2 (pol, AB517983); Bat betaherpesvirus B7D8, MsHV (cg, JQ805139); Caviid herpesvirus 2, CavHV2 (cg, NC_011587); *Rhinolophus ferrumequinum* herpesvirus 1, RfBHV1 (pol, JQ814845); Suid herpesvirus 2, SuHV2 (cg, NC_022233); Tupaiid herpesvirus 1, TuHV1 (cg, NC_002794); *Tylonycteris robustula* herpesvirus 1, TrBHV1 (pol, JQ814846). **(vi) Genus Lymphocryptovirus:** Callitrichine herpesvirus 3, CalHV3 (cg, NC_004367); Human herpesvirus 4, HHV4 (cg, V01555); Macacine herpesvirus 4, McHV4 (cg, NC_006146 ); Papiine herpesvirus 1, PaHV1 (pol, AY174069; ter, AF091052). **(vii) Genus Macavirus:** Alcelaphine herpesvirus 1, AlHV1 (cg, NC_002531); Alcelaphine herpesvirus 2, AlHV2 (cg, NC_024382); Bovine herpesvirus 6, BoHV6 (cg, NC_024303); Caprine herpesvirus 2, CpHV2 (ter, AF327834); Ovine herpesvirus 2, OvHV2 (cg, NC_007646); Suid herpesvirus 3, SuHV3 (pol and ter, AF478169); Suid herpesvirus 4, SuHV4 (pol and ter, AY170317); Suid herpesvirus 5, SuHV5 (pol and ter, AY170316). **(viii) Genus Percavirus:** Equid herpesvirus 2, EHV2 (cg, NC_001650); Equid herpesvirus 5, EHV5 (cg, NC_026421); Mustelid herpesvirus 1, MusHV1 (ter, AF275656). **(ix) Genus Rhadinovirus:** Ateline herpesvirus 3; AtHV3 (cg, NC_001987); Bovine herpesvirus 4, BoHV4 (cg, NC_002665); Cricetid herpesvirus 2, RHVP (cg, NC_015049); Human herpesvirus 8, HHV8 (cg, NC_009333); Macacine herpesvirus 5, McHV5 (cg, NC_003401); Murid herpesvirus 4; MuHV4 (cg, NC_001826); Murid herpesvirus 7, MuHV7 (cg, GQ169129); Saimiriine herpesvirus 2, SaHV2 (cg, NC_001350). **(x) Unassigned species in the Gammaherpesvirinae subfamily:** American bison gammaherpesvirus, bisonGHV (ter, AY057987); Asinine herpesvirus 4, AsHV4 (ter, AF535210); Asinine herpesvirus 5, AsHV5 (ter, AF535207); Asinine herpesvirus 6, AsHV6 (ter, AF535208); Callitrichine herpesvirus 1, CalHV1 (ter, AF091070); Deer malignant catarrhal fever virus, DHV (ter, AY055731); Leporid herpesvirus 2, LeHV2 (ter, AF091069); *Macaca fuscata* rhadinovirus, JMRV (cg, JN885137); *Macaca nemestrina* rhadinovirus 2, MneRV2 (cg, KP265674); *Macaca nemestrina* retroperitoneal fibromatosis-associated herpesvirus, RFHVMn (cg, KF703446); Otariid herpesvirus 1, OtHV1 (ter, AF236051).
