# Supplementary material for: Identification of Novel Betaherpesviruses in Iberian Bats Reveals Parallel Evolution
Source: PLoS One. 2016 Dec 30;11(12):e0169153. doi: 10.1371/journal.pone.0169153 (PMC5201282; doi:10.1371/journal.pone.0169153)

**Supporting information (S2)**

**Phylogenetic relationships of the potentially novel bat-hosted gammaherpesviruses**

The potentially novel gammaherpesviruses are represented in bold with their arrangement in relation to the main groups of gammaherpesviruses available in GenBank. The reconstruction was built under the Bayesian criterion allowing specific model rates. The consensus topology shows bayesian posterior probabilities (BPP) >0.7 after sampling 10^7^ generations.


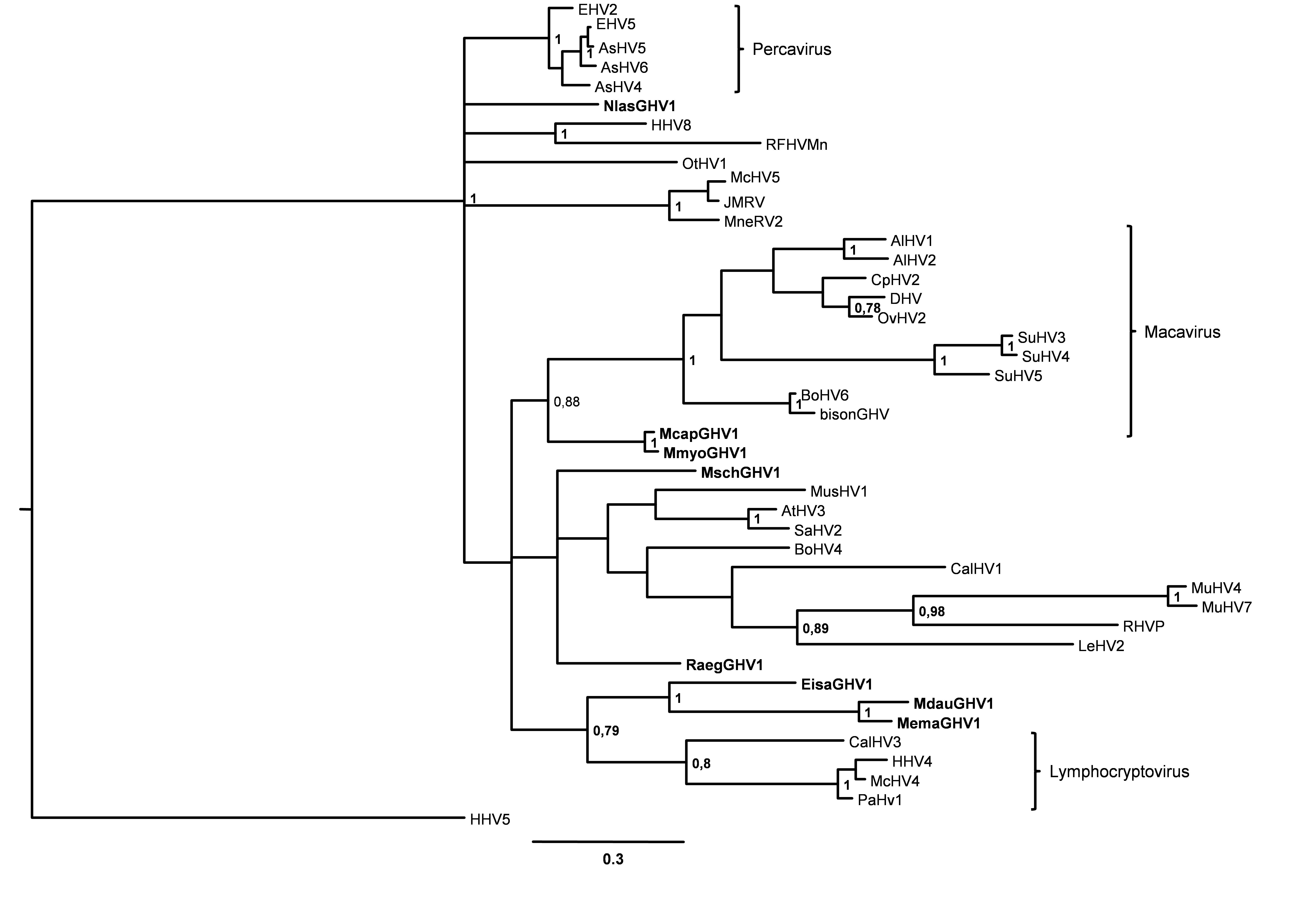

Supplement: S2 File — (DOCX) [file pone.0169153.s002.docx]
